# Image-Based RCS Estimation from Near-Field Data

**DOI:** 10.3390/jimaging5060061

**Published:** 2019-06-17

**Authors:** Tushar Rajvanshi, Maria Antonia Maisto, Angela Dell’Aversano, Raffaele Solimene

**Affiliations:** Department of Engineering, University of Campania, 81031 Aversa, Italy

**Keywords:** RCS estimation, image-based approach, adjoint inversion methods

## Abstract

This paper deals with the problem of estimating the RCS from near-field data by image-based approaches. In particular, a rigorous focusing procedure based on a weighted adjoint scheme, which is also applicable to an arbitrary measurement curve, is developed. The developed formalism allows us to address the important question concerning the need to employ a multi-frequency configuration to estimate the RCS. Accordingly, it is shown that if RCS is required at a given frequency, then the target image obtained solely at such a frequency can be exploited provided that the spatial truncation arising from the size of the investigated area is properly taken into account.

## 1. Introduction

The Radar Cross Section (RCS) of a target is a crucial quantity that describes how the target responds to an impinging electromagnetic wave (i.e., how it scatters the incident wave) across different directions. As is well known, RCS is formally defined as the distance between the probing antenna and the target approaching infinity [1]. In practical cases, to measure the RCS, the physical separation between the target and the illuminating/measuring antenna is required to comply with the far-field conditions. However, as frequency increases, and depending on the size of the target, the required separation can soon become very large. Therefore, for a long time, there has been a great interest in developing methods for predicting the RCS for scattered field measurements taken at a distance not in far-field [2,3,4,5,6]. Indeed, the possibility of acquiring data in near-zone avoids the above-mentioned drawback and in principle offers several advantages since measurements can be taken within an anechoic chamber. On the other hand, near-zone measurements cannot be used straight away for RCS estimation, due to the wavefront curvature and because in near-zone the target is not uniformly illuminated. Compact range equipment solves these problems, but requires high-quality reflectors [4,6]. Alternatively, image-based approaches first obtain a reconstruction of the target reflectivity and then the RCS is estimated by Fourier, transforming the obtained reflectivity. Accordingly, those approaches can be regarded as particular near-field to far-field transformations [7]. One can consider compact range methods and image-based approaches to move the job to do from hardware, i.e., the reflector, to software, i.e., the scattered field data processing algorithms.

In this paper, we are concerned with image-based approaches, in particular under a monostatic measurement setup.

The underlying working assumption is that the target scatters linearly, i.e., multiple scattering, shadowing, and creeping waves are considered negligible. Accordingly, the scattering phenomenon can be described by a linear integral scattering operator and the reconstruction of the target reflectivity is usually obtained by solving (inverting) that integral equation via some focusing procedure borrowed from back-propagation/migration algorithms literature [8].

Focusing entails some smearing in the reconstruction (depending on the measurement configuration parameters such as frequency band, etc.) which can negatively impact the subsequent RCS estimation. To compensate for such an effect, the obtained RCS can be normalized by the one corresponding to a point-like target [9] or, alternatively, the standard focusing kernel can be corrected by a suitable factor [10].

The first procedure implicitly assumes a spatially invariant behavior (within the image region) of the imaging procedure which indeed does not hold. The second approach has instead been demonstrated only for a circular measurement curve. The first contribution of this paper is the derivation of the focusing procedure under a general framework as far as the measurement curve is concerned, which actually generalizes the procedure reported in [10] to a generally shaped measurement curve.

To obtain high-resolution target reconstructions, the imaging stage is usually achieved by using multi-frequency data. However, target reflectivity in general depends on frequency. As outlined in [7], in that case, the obtained reflectivity can be considered more like an average over the employed frequency band. Furthermore, the obtained reflectivity reconstruction is generally used for RCS estimation just at the central frequency. It is then natural to ask if the imaging procedure can work by directly using single-frequency data.

This is indeed possible [11] even though RCS estimation is more sensitive to the spatial truncation determined by the size of the image region.

## 2. Basics on Image-Based RCS

In this section, we introduce the problem, the adopted notation, and briefly recall image-based methods present in literature.

**Figure 1:**
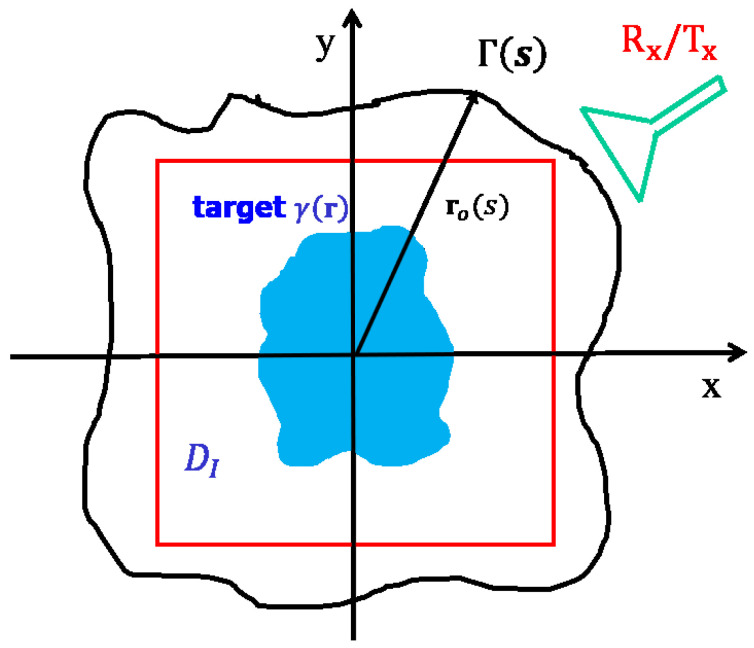
Geometry of the problem.

The scattering experiment related to the RCS estimation considered in this paper is described in Figure 1. In particular, we refer to a 2D scalar configuration, i.e., the target is invariant along the *z*-axis and the probing field is a cylindrical wave linearly polarized again along *z*. Accordingly, the scattered field is also linearly polarized and the RCS is a single scalar and not a dyad, as in the general 3D vector case. The target is assumed to reside within the *image* domain $D_{I}$, whereas the scattered field is collected in near-field over a curve Γ surrounding $D_{I}$ and for the frequency band Ω. The usual monostatic configuration is considered. Accordingly, during the data-acquisition stage, the same antenna acts as transmitter and receiver and then moves around the target to synthesize the measurement curve, or equivalently the target is rotated over a turntable. In the latter case the synthesized measurement curve is just a circle, which is the one that is usually considered in literature.

It is worth remarking that we are considering such a 2D configuration for the sake of simplicity, since this allows easier production of the numerical examples to be shown later on. However, a similar configuration is commonly addressed also in 3D cases while estimating the so-called *water line* RCS [7].

The starting point for problem formulation is the assumption that the target consists of an ensemble of independent and non-directional scattering centers [9]. Since multiple interactions, creeping waves and shadowing effects are neglected, the target reflectivity γ(r) and the scattered field are linked by the following linear integral operator

(1) $$E_{S}\left(k,r_{o}\left(s\right)\right)=\int_{D_{I}}A\left(k,r_{o}\left(s\right),r\right)e^{-j\phi [k,r_{o}\left(s\right),r]}\gamma \left(r\right)d^{2}r$$

where *k* is the wavenumber and $r_{o}\left(s\right)\in \Gamma$ is the measurement curve parametrized with respect to *s*. Moreover, the phase term takes into account the propagation path from the antenna to the target and back, i.e.,

$$\phi \left(k,r_{o},r\right)=2k\left|r_{o}\left(s\right)-r\right|$$

whereas the amplitude term is given by

$$A\left(k,r_{o}\left(s\right),r\right)=\frac{\alpha C\left(k\right)P^{2}\left[\theta (r_{o}\left(s\right),r)\right]}{|r_{o}\left(s\right)-r|}$$

with α being a constant term that varies depending on whether a 3D or 2D geometry is being considered, $P[\theta (r_{o}\left(s\right),r)]$ is the antenna directivity pattern assumed to be 1 for $\theta (r_{o}\left(s\right),r)=0$, (with $\theta (r_{o}\left(s\right),r)$ being the angle between the $r_{o}\left(s\right)-r$ and the antenna broadside direction) and C(k) collects the antenna frequency behavior and the frequency part of the propagator (Green function) amplitude. In particular, for the case at hand, the relevant Green function is proportional to a Hankel function of zero order and second kind. $P[\theta (r_{o}\left(s\right),r)]$ and C(k) are assumed to be known by a preliminary stage of measurement and calibration using a reference target of known RCS such as a metallic sphere, a plate or the like.

At this juncture, some further considerations concerning the model (1) are in order. First, it is noted that in (1) each single point in the image domain has been considered to be being in the far-field of the transmitting/receiving antenna. If this is not the case, a linear transformation could be still established by invoking the plane-wave spectrum representation for both the Green function and the antenna response. This circumstance is not considered in this manuscript. Second, field data, instead of voltage, are being considered. Voltage is actually what one can measure. However, assuming field data does not impair the generality of the theoretical/numerical analysis we intend to pursue. Finally, the target reflectivity is in general frequency dependent; we denote such a dependence by $\gamma_{f}\left(k\right)$. However, if the target can be represented by an ensemble of point-like scatterers, the frequency dependent part of reflectivity is a priori known and hence can be considered embodied within the C(k) term [10]. However, we will turn back on this assumption in the following sections.

The near-field (1) is in general much different from the corresponding far-field and hence cannot be used directly for estimating the target RCS. This of course is due to the wavefront curvature of the impinging electromagnetic wave and to the non-uniform illumination of the target. The aim of the imaging stage is just to compensate for such wavefront curvature and amplitude behavior. This is achieved by processing the scattered field data through a focusing operator that actually “translates” the scattered field data into an image of the target under test. Formally, this is written as

(2) $$\tilde{\gamma }\left(r\right)=\int_{\Omega \times \Gamma }f_{c}\left[k,r_{o}\left(s\right),r\right]E_{S}\left[k,r_{o}\left(s\right)\right]dkds$$

where $f_{c}\left[k,r_{o}\left(s\right),r\right]$ is the focusing kernel which is commonly chosen equal to $e^{j\phi [k,r_{o}\left(s\right),r]}/A\left[k,r_{o}\left(s\right),r\right]$. Clearly, $\tilde{\gamma }$ is a filtered (blurred) version of the actual reflectivity. That filtering depends on the configuration parameters, i.e., Ω×Γ, which reflect the properties of the imaging point-spread function, i.e.,

(3) $$psf\left(r,r^{\prime }\right)=\int_{\Omega \times \Gamma }\frac{A[k,r_{o}\left(s\right),r^{\prime }]}{A[k,r_{o}\left(s\right),r]}e^{j[\phi \left[k,r_{o}\left(s\right),r\right]-\phi \left[k,r_{o}\left(s\right),r^{\prime }\right]]}dkds$$

so that (2) can be equivalently rewritten as

(4) $$\tilde{\gamma }\left(r\right)=\int_{D_{I}}psf\left(r,r^{\prime }\right)\gamma \left(r^{\prime }\right)d^{2}r^{\prime }$$

Once the reflectivity has been estimated, the RCS can be computed by using its (2D) definition equation

(5) $$\sigma \left(k,\theta_{o}\right)=\lim_{r_{o}\to \infty }2\pi r_{o}{\left|\frac{E_{S}\left(k,r_{o}\right)}{E_{inc}}\right|}^{2}$$

with $r_{o}\equiv \left(r_{o},\theta_{o}\right)$ being the observation point. Hence, on exploiting (1) and (2), (5) yields

(6) $$\sigma \left(k,\theta_{o}\right)={\left|\alpha \gamma_{f}\left(k\right)\int_{D_{I}}\tilde{\gamma }\left(r\right)e^{2jk{\hat{r}}_{o}\cdot r}d^{2}r\right|}^{2}$$

Equation (6) allows recognition that under the assumed linear scattering model, the RCS depends of the Fourier transform of the reflectivity function projected over the so-called Ewald disc (resp. sphere for the 3D case) [12]. Of course, because of the blurring introduced by the imaging procedure, some error will corrupt the estimation (6). In order to mitigate such an error, a common way to proceed is to normalize (6) by the RCS of the point-spread function [9], which is computed by Fourier transforming psf(r,0). It is clear that behind this procedure there is the implicit assumption of considering (4) as a convolution which in general does not hold. Differently, in [10], it is shown that by introducing a suitable correction term, the focusing procedure can be approximated as a Fourier transformation. Accordingly, $\tilde{\gamma }$ proves to be a windowed (in spatial spectrum domain) Fourier transform of the γ; therefore, normalization is no longer required.

We will come back to these important points in the next section where we introduce an imaging procedure for a more general (with respect to the usual circle) measurement curve.

## 3. Adjoint Inversion for Generic Measurement Curve

According to the previous section, the first step in any image-based RCS estimation method is to solve the integral Equation (1) for the reflectivity γ. Formally, this entails finding the inverse of the linearized scattering operator

(7) $$A_{S}:\gamma \to E_{S}$$

$A_{S}$ being just the integral operator in (1). A very common way to invert (7) is to adopt the adjoint operator $A_{S}^{†}$ instead of the inverse of $A_{S}$. A number of popular methods such as time-reversal, reverse-migration, back-propagation, etc. are adjoint-based imaging schemes [8]. Inversion through the adjoint allows us to deal with the ill-posedness of the problem in that it retunes a stable reconstruction procedure. However, it is not a Tichonov regularization scheme as the corresponding reconstruction fails to converge to the generalized solution even in absence of noise [8]. Their great diffusion is due to their simple *physical* understanding and the possibility to be often implemented via FFT. In particular, adjoint inversion succeeds in compensating the phase in correspondence to the actual scatterers’s position but the amplitude is not addressed properly. For this reason, adjoint inversion is often paired with a pre-weighting (filtering) stage, so that the image/reconstruction is obtained as [13]

(8) $$A_{S}^{†}:WE_{S}\to \tilde{\gamma }$$

where $W(k,r_{o},r)$ is just the weighting function. In particular, on exploiting (1), the reconstruction operator (8) can be explicitly written as

(9) $$\tilde{\gamma }\left(r\right)=\int_{\Omega \times \Gamma }W\left[k,r_{o}\left(s\right),r\right]e^{j\phi [k,r_{o}\left(s\right),r]}E_{S}\left[k,r_{o}\left(s\right)\right]dkds$$

which yields the point-spread function

(10) $$psf\left(r,r^{\prime }\right)=\int_{\Omega \times \Gamma }W\left[k,r_{o}\left(s\right),r\right]A\left[k,r_{o}\left(s\right),r^{\prime }\right]e^{j\{\phi \left[k,r_{o}\left(s\right),r\right]-\phi \left[k,r_{o}\left(s\right),r^{\prime }\right]\}}dkds$$

Please note that the usual choice for the weighting function, as done in the previous section, is to $W\left[k,r_{o}\left(s\right),r\right]=1/A\left[k,r_{o}\left(s\right),r\right]$. On the other hand, it is desirable that the point-spread function be as close as possible to a delta function. If this is achieved, the adjoint inversion actually approximates a regularized reconstruction [14]. To cope with this question, the weighting function must be properly chosen as detailed below.

We start by introducing the variable

(11) $$w\left[k,r_{o}\left(s\right),r,r^{\prime }\right]=-\int_{0}^{1}\nabla_{x}\phi \left[k,r_{o}\left(s\right),x\right]{|}_{x=r^{\prime }+\lambda \left(r-r^{\prime }\right)}d\lambda$$

which allows rewriting of the phase term in (10) as

(12) $$\phi \left[k,r_{o}\left(s\right),r\right]-\phi \left[k,r_{o}\left(s\right),r^{\prime }\right]=-w\left[k,r_{o}\left(s\right),r,r^{\prime }\right]\cdot \left(r-r^{\prime }\right)$$

and the point-spread function (10) as

(13) $$psf\left(r,r^{\prime }\right)=\int_{\Omega_{w}}W\left(w,r\right)A\left(w,r^{\prime }\right)e^{-jw\cdot (r-r^{\prime })}\left|J\right|d^{2}w$$

with J being the Jacobian of the transformation that maps w in (k,s). Equation (13) shows the point-spread function as pseudodifferential operators which enjoy the so-called pseudolocal property [15]. This is the very mathematical rationale that justifies performing the adjoint inversion to retrieve object singularities, even though in order to correctly retrieve the singularity amplitudes the weighting function must be properly chosen. Also, (13) makes it immediately clear that the leading order contribution occurs for $r-r^{\prime }=0$. Accordingly, the approximation

(14) $$w\left[k,r_{o}\left(s\right),r,r^{\prime }\right]=w\left[k,r_{o}\left(s\right),r,r\right]=-\nabla_{x}\phi \left[k,r_{o}\left(s\right),x\right]{|}_{x=r}$$

is made in (13). This leads to the following point-spread function approximation

(15) $$psf\left(r,r^{\prime }\right)≃\int_{\Omega_{w}}e^{-jw\cdot (r-r^{\prime })}d^{2}w$$

once the weighting function is chosen as

(16) $$W\left(w,r\right)=1/\left[A\left(w,r\right)\left|J\right|\right]$$

It is seen that (15) is a filtered version of a Dirac delta. Moreover, Equation (15) makes it clear the spectral content of the target that can be retrieved. Indeed, when the observation curve Γ goes around the target it results that

(17) $$\Omega_{w}=\left\{w:2k_{min}\leq w\leq 2k_{max}\right\}$$

where $k_{min}$ and $k_{max}$ are the wavenumbers corresponding to the lowest and highest adopted frequencies. This result could have been expected since it exactly coincides to what can be retrieved by a far-field configuration. However, it must be kept in mind that (17), and of course (15), holds only approximately true because of the approximation (14). However, (14) is expected to work well in reproducing the main beam of the point-spread function, which in turn plays the major role in the filtering introduced by the imaging procedure.

**Figure 2:**
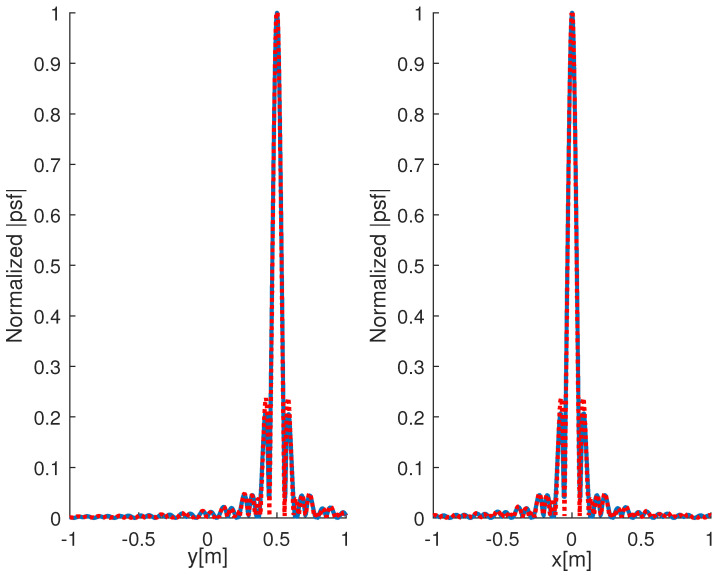
Comparison between the normalized point-spread function amplitude (blue line) and the one returned by (18) (red dotted line). The left panel refers to the cut along the *y*-axis; the right one to the cut along the *x*-axis. The adopted frequency band is Ω=[0.5,1.5] GHz while the observation curve is an ellipse with axes of 2 m and 2.5 m, respectively.

It is interesting to highlight that (15) holds true regardless of the shape of the observation curve Γ. Indeed, the shape of Γ only enters in the choice of the weighting function through the Jacobian term. In particular, when Γ is a circle surrounding the image region (as it is commonly assumed) simple calculations show that (15) exactly returns the imaging kernel introduced in [10], with the correcting term there introduced just being given by 1/|J|. In this regard, the formulation introduced in this paper generalizes previous literature results, which indeed have mainly considered circular measurement curves. Previous discussion can be summarized by the following statement: the resolution achievable during the image stage does not depend on the shape of the measurement curve. Moreover, by exploiting techniques similar to those in [16], an analytical expression for the point-spread function can be obtained. In particular, it can be shown that the point-spread function in (15) is given as

(18) $$psf\left(r,r^{\prime }\right)≃\frac{2\pi [\psi (2k_{max}|r-r^{\prime }|)-\psi (2k_{min}|r-r^{\prime }\left|)\right]}{|r-r^{\prime }{|}^{2}}$$

with

(19) $$\psi \left(x\right)=\int_{0}^{x}yJ_{0}\left(y\right)dy=xJ_{1}\left(x\right)$$

and $J_{0}\left(\cdot \right)$ and $J_{1}\left(\cdot \right)$ being Bessel functions of zero and first order. In Figure 2, the comparison between the actual point-spread function and the one returned by (18) is shown. As can be appreciated, the two curves overlap very well, this means that the leading term approximation exploited to derive (18) works fine.

A further point that is worth stressing is that for a full-view scan (i.e., for a measurement curve running all around the image region) the spectral domain $\Omega_{w}$ does not depend on the image point r. This means that the image kernel (15) can be actually considered to be convolution; however, this does not hold true for (10), since to get (15) a spatially varying kernel was required. Accordingly, the normalization procedure in [9] can be expected to work only when the image points are close to the reference one (i.e., the one for which the point-spread function is computed and transformed).

Finally, it is remarked that to speed up the imaging procedure FFT-based routines are often employed [17]. According to the previous formulation, this can be achieved for a generic measurement curve. Of course, the usual interpolation and resampling step, which is required to map data over a uniform grid, in general depends on the particular curve under concern. In the following numerical examples we do not follow such a procedure, rather we employ (9) with the weighting function chosen as in (16).

## 4. Single-Frequency Imaging

As clearly stated above, the previous RCS estimation procedure is founded on the assumption that the targets are frequency independent or their frequency behaviors are equal and known. The latter holds true for point-like targets but for most practical cases, even when targets can be still described by an ensemble of scattering centers, the different contributions depend on the operating frequency. Accordingly, the reflectivity returned by (9) is actually an averaged version performed over the frequency band. This circumstance in general impacts negatively on the RCS computation. That is why, the obtained image is usually used to compute the RCS only at the central (of the adopted band) frequency [7].

Let us relax the above-mentioned assumption. Hence, here we consider the cases in which the target frequency behavior is unknown, or it consists of scattering centers whose reflectivity coefficients differently depends on frequency. In these cases, the frequency behavior cannot be singled out and embodied within the scattering operator since actually γ=γ(k,r). Accordingly, the scattered field is yielded by

(20) $$E_{S}\left[k,r_{o}\left(s\right)\right]=\int_{D_{I}}A\left[k,r_{o}\left(s\right),r\right]e^{-j\phi [k,r_{o}\left(s\right),r]}\gamma \left(k,r\right)d^{2}r$$

and the estimated reflectivity (through imaging) and the actual one are linked as

(21) $$\tilde{\gamma }\left(r\right)=\int_{\Omega }dk\overset{\tilde{\gamma }\left(k,r\right)}{\overset{︷}{\int_{D_{I}}\int_{\Gamma }f_{c}\left[k,r_{o}\left(s\right),r\right]A\left[k,r_{o}\left(s\right),r^{\prime }\right]e^{-j\phi [k,r_{o}\left(s\right),r^{\prime }]}\gamma \left(k,r^{\prime }\right)dsd^{2}r^{\prime }}}$$

It is seen that $\tilde{\gamma }\left(r\right)$ is now given as the coherent summation of the single-frequency images $\tilde{\gamma }\left(k,r\right)$ and as such it does not represent the (regularized) solution of (20). This could have been expected since both scattered field data and unknown reflectivity depend on the frequency. As discussed above, this leads to a degradation of accuracy while estimating the RCS.

The arising question is whether, in order to estimate the RCS at a given frequency, a frequency band must necessarily be employed. The answer to this question seems positive if high-resolution radar imaging is aimed. At the other hand, single-frequency images are not affected by previous drawback but the performance in the reconstruction in general results much lower. Hence, the question can be rephrased as whether image degradation (due to single-frequency data) impairs RCS estimation. To this end, we particularize (15) to the single-frequency case, for example by considering $k=k_{av}$ (i.e., the average one)

(22) $$psf_{sf}\left(r,r^{\prime }\right)≃\int_{C_{w}}e^{-jw\cdot (r-r^{\prime })}d^{2}w$$

where the subscript sf stands for single-frequency and $C_{w}=\left\{w:w=2k\right\}$. (22) can be easily computed and the result is

(23) $$psf_{sf}\left(r,r^{\prime }\right)≃4\pi kJ_{0}(2k|r-r^{\prime }\left|\right)$$

**Figure 3:**
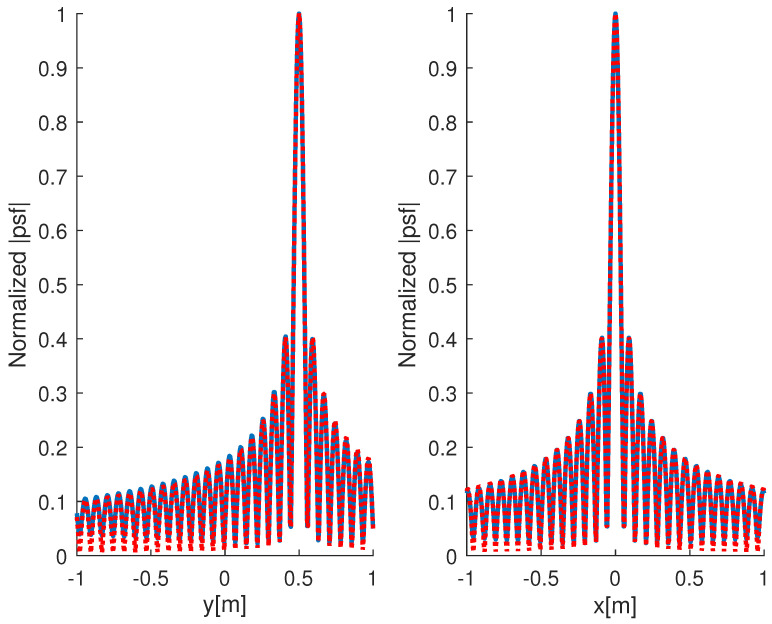
Comparison between the normalized point-spread function amplitude (blue line) and the one returned by (23) (red dotted line). The left panel refers to the cut along the *y*-axis whereas the right one to the cut along the *x*-axis. The frequency is 1 GHz while the observation curve is an ellipse with axes 2 m and 2.5 m.

In Figure 3 the comparison between the actual point-spread function and the one returned by (23) is shown. Again, the estimated psf allows us to obtain a good approximation of the actual one.

To understand how resolution degrades when single-frequency data are used, Equation (23) can be compared to (18). It is clear that now the spatial spectrum that can be retrieved about the unknown reflectivity is much lower since $C_{w}$ consists of a single circle instead of the circular annulus $\Omega_{w}$. Nonetheless, as shown in [16], it is the relative bandwidth (i.e., $\left(k_{max}-k_{min}\right)/k_{av}$), rather than the frequency band, that plays the major role. This is somewhat different from the common belief that the frequency band is necessary and is a consequence of the full-view (i.e., measurements are taken all around the target) configuration. However, we do not want to dwell any further on that point since this is not the focus of the paper. The point is whether image degradation impairs the possibility of obtaining RCS. To this end, we once again remark that (22) introduces a filtering that allows retention of only the target reflectivity over the circle $C_{w}$, which is the Ewald circle corresponding to the wavenumber *k* and which is, according to (6), what is necessary to compute the RCS.

Finally, by looking at Figure 3, it can be seen that the side-lobes are higher than the multi-frequency case. This allows expectation that while comparing multi-frequency and single-frequency RCS estimations, the spatial truncation introduced by the size of the investigated area will play a crucial role.

**Figure 4:**
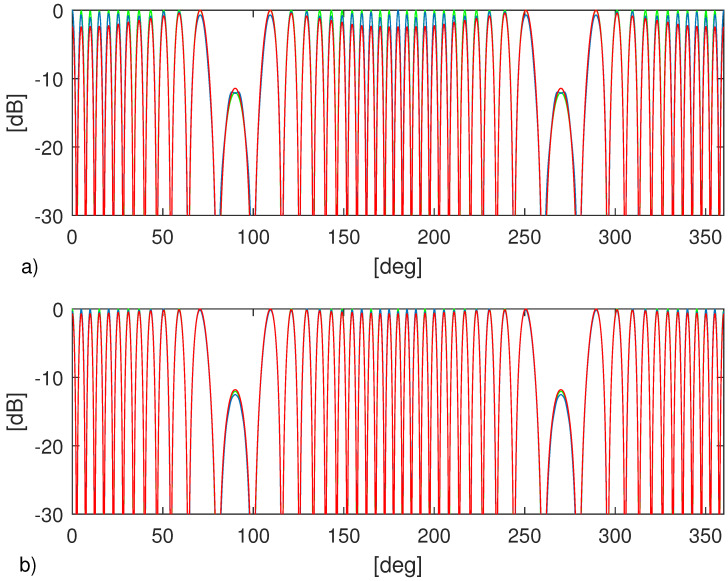
Normalized RCS (in db scale) at f=1.75 GHz. The green lines refer to the actual RCS, while the blue and red lines to the ones computed from the multi-frequency and single-frequency images, respectively. (**a**) $D_{I}=\left[\left(-1,1\right)\times \left(-1,1\right)\right]$ and (**b**) $D_{I}=\left[\left(-2,2\right)\times \left(-2,2\right)\right]$.

To check previous arguments, we conclude this section by running a simple case. In particular, we consider two point-like objects whose reflectivity is given by $\gamma \left(x,y\right)=0.1{\left(j\frac{k}{k_{min}}\right)}^{2}\left[\delta (x,y-0.5)+\delta (x,y+0.5)\right]$. The electric field is collected over an ellipse whose axes are 3 m and 3.5 m and the working frequency is f=1.75 GHz. Since the far-field distance is 11.7 m, the ellipse lies in the near-field region of two point scatterers. In Figure 4 the RCS at that frequency is shown. The green lines refer to the actual RCS, while the blue and red lines to the ones computed from the multi-frequency (Ω=[0.5,3]GHz) and single-frequency images, respectively. In particular, in the panel a) $D_{I}=\left[\left(-1,1\right)\times \left(-1,1\right)\right]$ is considered whereas for panel b) $D_{I}=\left[\left(-2,2\right)\times \left(-2,2\right)\right]$. From the Figure 4a) it can be appreciated that the multi-frequency configuration allows us to obtain a better RCS estimation. However, when $D_{I}$ increases (see Figure 4b) the red line becomes very similar to the blue one. This confirms that single-frequency RCS is feasible and competitive with respect to the multi-frequency one provided the size of the investigated area is enlarged.

## 5. Numerical Examples

In this section, some further examples are addressed to check RCS estimation.

**Figure 5:**
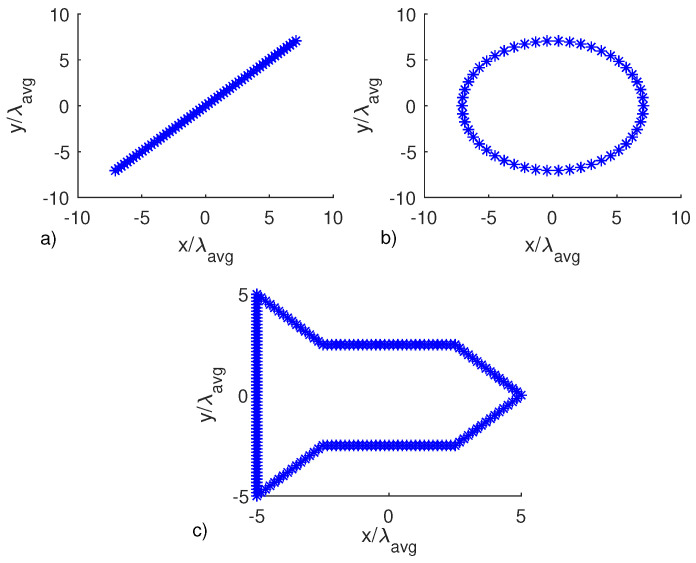
Contours of the objects under test.

In all the following examples, the RCS is evaluated at $f_{avg}=4.5$ GHz, the field is collected over an ellipse whose axes are $30\lambda_{avg}$ and $35\lambda_{avg}$ with $\lambda_{avg}$ the wavelength at $f_{avg}$ and $D_{I}=\left[\left(-20\lambda_{avg},20\lambda_{avg}\right)\times \left(-20\lambda_{avg},20\lambda_{avg}\right)\right]$. Moreover, when the multi-frequency configuration is exploited Ω=[3,6] GHz. The target, always a perfect electric conducting scatterer, is contained within a circular image region with radius $5\sqrt{2}\lambda_{avg}$. Accordingly, the far-field distance is $400\lambda_{avg}$ and the ellipse lies in the near-field region of the object. Three different objects, shown in Figure 5, are considered.

**Figure 6:**
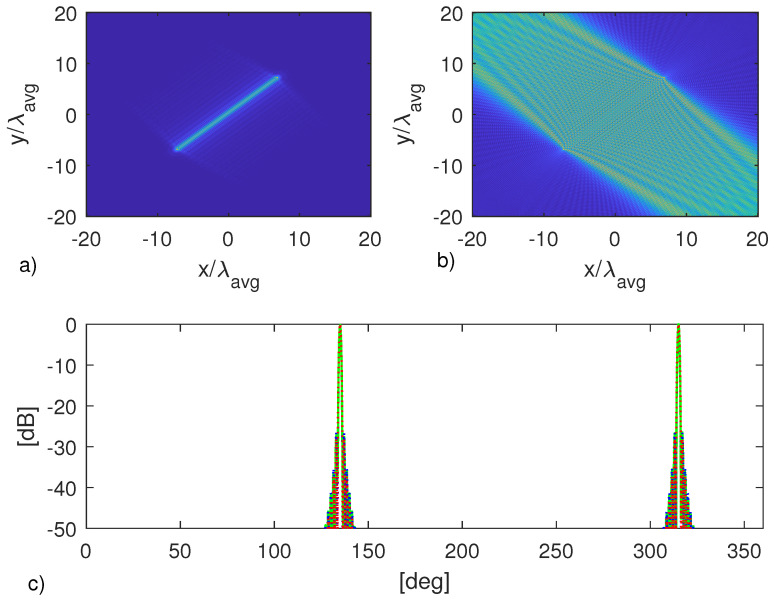
Object in Figure 5a. (**a**) Multi-frequency image. (**b**) Single-frequency image. (**c**) Normalized RCS at f=4.5 GHz. The green line refers to the actual RCS, while the blue and red lines to the ones computed from multi-frequency and single-frequency images, respectively.

The first object is a bar of length $10\sqrt{2}\lambda_{avg}$. The corresponding multi-frequency and single-frequency images are reported in the panels (a) and (b) of Figure 6, while the RCS estimations are compared in (c). As can be seen, despite the evident degradation of the reconstructed image, single-frequency configuration allows us to obtain an RCS estimation which is very similar to the multi-frequency one; indeed the three curves overlap very well.

**Figure 7:**
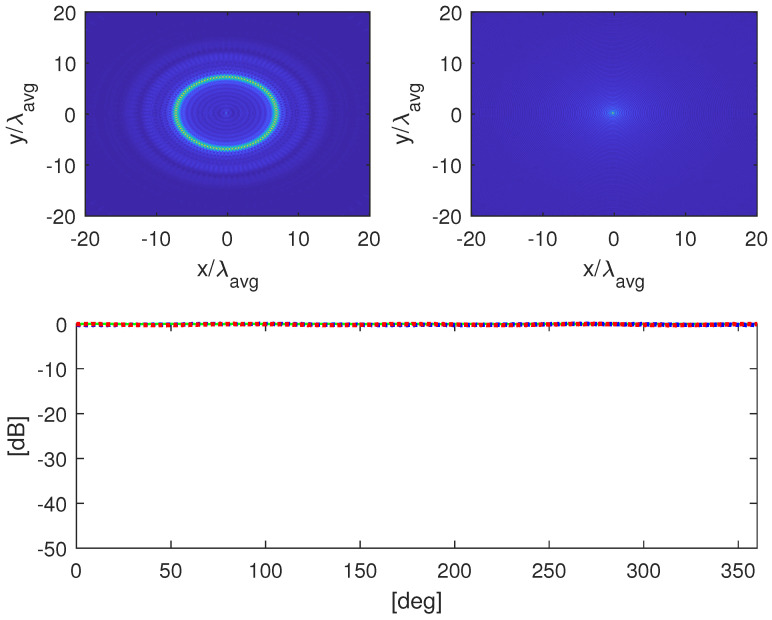
Object in Figure 5b. (**a**) Multi-frequency image. (**b**) Single-frequency image. (**c**) Normalized RCS at f=4.5 GHz. The green line refers to the actual RCS, while the blue and red lines to the ones computed from the multi-frequency and single-frequency images, respectively.

The circular object (of radius $5\sqrt{2}\lambda_{avg}$) is addressed in Figure 7. As can be seen, the multi-frequency reconstruction allows us to clearly discern the shape of the target which is completely lost in the single-frequency case. Nonetheless. the RCS estimation is comparable with the multi-frequency one.

**Figure 8:**
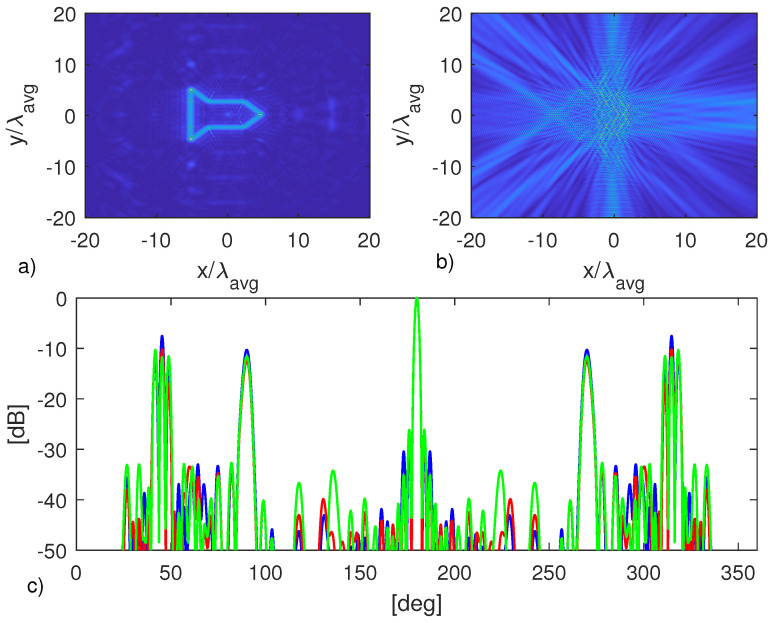
Object in Figure 5c. (**a**) Multi-frequency image. (**b**) Single-frequency image. (**c**) Normalized RCS at f=4.5 GHz. The green line refer to the actual RCS, while the blue and red lines to the ones computed from the multi-frequency and single-frequency images, respectively.

The last considered example, the missile shaped target is addressed in Figure 8. The same discussion as for the previous examples still holds. In this case, it must be remarked that even though the main RCS features (spikes) are captured, both the multi-frequency and the single-frequency approaches show some deviation from the actual RCS. This can be ascribed to the model error (for example multiple scattering, which is more relevant for this complex shaped target) that inherently affects image-based procedure.

## 6. Conclusions

In this paper, the problem of estimating the RCS from near-field data by image-based approaches has been addressed.

The first contribution we conveyed in this manuscript was the rigorous derivation of a focusing procedure based on a weighted adjoint scheme which generalizes for an arbitrary measurement curve the results presented in [10] that have been developed for a circular measurement curve.

Secondly, the important question concerning the necessity to use a multi-frequency configuration to estimate the RCS has been studied. Accordingly, a deep analysis of the introduced analytical tools highlighted that if RCS is required at a given frequency, then the target image obtained solely at such a frequency can be in principle exploited. This opens the possibility of employing cheaper measurement systems and to take into account target frequency dependence. However, the spatial truncation introduced by the size of the investigated area must be properly taken into account.

Several numerical examples corroborated the possibility of computing the RCS from single-frequency image.

For the sake of simplicity, the study has been developed for a 2D scalar configuration. The generalization to 3D real scenario is a possible future development.
